# Genome Sequences of Three Cluster C Mycobacteriophages, Bipolarisk, Bread, and FudgeTart

**DOI:** 10.1128/MRA.00290-19

**Published:** 2019-07-11

**Authors:** Dane M. Bowder, Brandon W. Gannon, Kathryn J. Grint, Jason T. Iltz, Teryn M. Koch, Kaitlyn S. Mahnke, Brea D. Murnan, Alexandria M. Osborn, Danielle M. Schreiber, Grace L. Su, Jaime G. Troester, Erin L. Doyle

**Affiliations:** a Department of Biology, Doane University, Crete, Nebraska, USA; Queens College

## Abstract

Three mycobacteriophages, Bipolarisk, Bread, and FudgeTart, were isolated from enriched soil samples found in Crete, NE. All three phages are lytic, belong to subcluster C1, and infect Mycobacterium smegmatis mc^2^155. The structures of the three genomes are similar, with slight variations in gene number and content.

## ANNOUNCEMENT

Bacteriophages have the potential to be used therapeutically against bacterial pathogens (1). Mycobacteriophages are viruses that specifically infect and lyse mycobacterial hosts (2). Three phages, Bipolarisk, Bread, and FudgeTart, were isolated from soil samples collected on the Doane University campus in Crete, NE. Samples were enriched with Mycobacterium smegmatis mc^2^155 in 7H9 broth at 37°C. Phages were purified and amplified by growth on 7H9 medium with the host. All three are lytic and classified as subcluster C1 mycobacteriophages. Each phage is a myovirus, a trait shared with other cluster C phages (2). Genomic DNA was extracted from lysates using the Wizard DNA extraction kit (Promega).

DNA was sequenced at The Pittsburgh Bacteriophage Institute. Sequencing libraries were prepared from genomic DNA using an NEB Ultra II kit with dual-indexed barcoding. Libraries were pooled and run on an Illumina MiSeq instrument, yielding at least 100,000 single-end 150-base pair reads and at least 600-fold coverage for each genome. No further quality control or adapter trimming was performed on the reads provided by the sequencer. These raw reads were assembled using Newbler v.2.9 (default settings). Each genome yielded a single contig, which was checked for completeness, accuracy, and phage genomic termini using Consed v.29 as previously described (3). Gene start sites were predicted using GLIMMER v.3.02 (4) and GeneMark v.2.8 (5) using default settings and manually checked for appropriate gaps/overlaps, ribosome binding site (RBS) scores, and BLAST alignments to related phages using DNA Master v.5.23.2. (http://cobamide2.bio.pitt.edu/computer.htm). tRNAs were identified using ARAGORN v.1.1 (6) and tRNAscan-SE v.2.0 (6) using default settings. Predicted gene functions were assigned using NCBI BLAST (7), PECAAN (8), PhagesDB.org (9), Starterator v.1.1 (https://seaphages.org/software/), HHPRED v.2.0.16 (10), and Phamerator (11).

The genome lengths for Bipolarisk, Bread, and FudgeTart range from 153,796 bp to 154,734 bp, with G+C contents ranging from 64.7% to 64.8% (Table 1), similar to the host bacterium (5). All three phages have a similar number of genes with predicted functions and tRNAs (Table 1), with each containing one transfer-messenger RNA (tmRNA). The phage genomes are highly similar, with greater than 99.4% nucleotide identity and coverage of 96% to 97%, determined by nucleotide BLAST.

**TABLE 1:** Characteristics of Bipolarisk, Bread, and FudgeTart

Phage name	GenBank accession no.	SRA accession no.	Approx shotgun coverage (×) (no. of reads)	Genome size (bp)	No. of genes	No. of genes with predicted functions	G+C content (%)	No. of tRNAs
Bipolarisk	MK450429	SRX4877857	653 (743,920)	154,734	231	58	64.7	33
Bread	MH779498	SRX4877856	600 (653,424)	153,796	230	60	64.8	31
FudgeTart	MH779502	SRX4877858	621 (680,367)	154,658	231	61	64.8	31

The genomes of Bipolarisk, Bread, and FudgeTart are circularly permuted and canonically arranged (9), with the majority of binding and attachment genes colocated near the 5′ end of the annotated sequence, structural and viral assembly genes in the middle, and lysis cassette genes near the 3′ end. Despite structural similarities, there are a few notable differences in specific genes between the three genomes, such as FudgeTart_104, which differs from Bipolarisk_104 and Bread_105. The amino acid sequence for this gene in Bipolarisk and Bread is 54% identical to the gene in FudgeTart at the same position. This gene in FudgeTart is rarely called in cluster C phages and is present in all currently known cluster DO phages, which infect Gordonia terrae. Additionally, FudgeTart does not contain the gene directly upstream (Bipolarisk_103 and Bread_104).

### Data availability.

Genome sequences and raw sequence data are available in GenBank and the Sequence Read Archive. Accession numbers are shown in Table 1.
